# Downregulation of TET1 Promotes Bladder Cancer Cell Proliferation and Invasion by Reducing DNA Hydroxymethylation of AJAP1

**DOI:** 10.3389/fonc.2020.00667

**Published:** 2020-05-21

**Authors:** Yi-lin Yan, Zheng-nan Huang, Zhen Zhu, Yang-yan Cui, Mei-qian Li, Rui-min Huang, Jun Yan, Bing Shen

**Affiliations:** ^1^Department of Urology, Shanghai General Hospital, Shanghai Jiaotong University School of Medicine, Shanghai, China; ^2^Model Animal Research Center of Nanjing University, Nanjing, China; ^3^Shanghai Institute of Materia Medica, Chinese Academy of Sciences, Shanghai, China; ^4^University of Chinese Academy of Sciences, Beijing, China; ^5^Department of Laboratory Animal Science, Fudan University, Shanghai, China

**Keywords:** ten-eleven translocation 1 (TET1), DNA methylation, bladder cancer, vitamin C, adherens junction-associated protein 1 (AJAP1)

## Abstract

Ten-eleven translocation 1 (TET1) is a member of methylcytosine dioxygenase, which catalyzes 5-methylcytosine (5 mC) to 5-hydroxymethylcytosine (5 hmC) to promote the demethylation process. The dysregulated TET1 protein and 5 hmC level were reported to either suppress or promote carcinogenesis in a cancer type-dependent manner. Currently, the role of TET1 in the development of urinary bladder cancer (UBC) and its underlying molecular mechanisms remain unclear. Herein, we found that TET1 expression was downregulated in UBC specimens compared with normal urothelium and was inversely related to tumor stage and grade and overall survival, suggesting its negative association with UBC progression. TET1 silencing in UBC cells increased cell proliferation and invasiveness while the ectopic expression of wild-type TET1-CD, but not its enzymatic inactive mutant, reversed these effects and suppressed tumorigenicity *in vivo*. In addition, as a direct regulator of TET1 activity, vitamin C treatment increased 5 hmC level and inhibited the anchorage-independent growth and tumorigenicity of UBC cells. Furthermore, we found that TET1 maintained the hypomethylation in the promoter of the AJAP1 gene, which codes for adherens junction-associated protein 1. The downregulation of AJAP1 reversed TET1-CD-induced nuclear translocation of β-catenin, thus inhibiting the expression of its downstream genes. In human UBC specimens, AJAP1 is frequently downregulated and positively associated with TET1. Notably, low expression levels of both TET1 and AJAP1 predict poor prognosis in UBC patients. In conclusion, we found that the frequently downregulated TET1 level reduces the hydroxymethylation of AJAP1 promoter and subsequently activates β-catenin signaling to promote UBC development. The downregulation of both TET1 and AJAP1 might be a promising prognostic biomarker for UBC patients.

## Introduction

Urinary bladder cancer (UBC) is among the major causes of cancer-related deaths worldwide and is one of the most common cancers in the urinary tract, with nearly 550,000 newly diagnosed cases of UBC and 200,000 deaths worldwide in 2018 (1). Though non-muscle-invasive bladder cancers, which account for ~75% of UBC cases, are often treatable but recur frequently, the rest 25% of UBCs are muscle-invasive with poor prognosis, despite optimal treatment with surgery and chemotherapy (2, 3). Therefore, it is of great significance to elucidate the molecular mechanisms that promote UBC development and progression.

The process of UBC progression is complex, including genetic and epigenetic abnormalities (4, 5). Recent studies demonstrated that hypermethylation of promoters and subsequently silenced tumor suppressor genes were involved in UBC development (6, 7). Ten-eleven translocation (TET) family members are key players of DNA demethylation contrary to DNA methyltransferases (DNMTs). They can maintain genes in an unmethylated state by acting as a dioxygenase *via* conversion of 5-methylcytosine (5 mC) to 5-hydroxymethylcytosine (5 hmC) or by competing with DNMTs which results in passive demethylation (8). Aberrant expression of TET1 was reported to be more frequently detected in solid tumors, while TET2 was frequently mutated in hematopoietic malignancy and TET3 was less mentioned (9). As a frequently downregulated gene, TET1 acts as a tumor suppressor in multiple malignancies such as breast, gastric, colon, nasopharyngeal, and renal cancer (10–14). However, in some other cancers such as ovarian and triple-negative breast cancer, TET1 can promote carcinogenesis. The evidences above suggest that TET1 functions in a cell context-dependent manner (15, 16). So far, the role of TET1 in UBC has not been clearly elucidated.

Abnormal activation of Wnt/β-catenin pathway has been implicated in human UBC progression (17). Once Wnt ligands bind to Frizzled (Fz)-low-density-lipoprotein (LRP) receptors, the complex induces stabilization and nuclear localization of β-catenin, which eventually coactivates transcription factor (TCF) to transactivate downstream target gene expression. We previously identified Wnt7A as a key positive regulator to activate the canonical Wnt/β-catenin pathway and subsequently to promote metastasis of UBC cells to the lung (18). On the contrary, there also exist several Wnt antagonists, which consist of secreted frizzled-related protein (sFRP) and Dickkopf (DKK) members (19). The sFRP proteins inhibit Wnt signaling by directly binding to Wnt proteins, while DKKs bind to the LRP5/LRP6 components of the Wnt receptor complex. In addition, a number of negative regulators of Wnt signaling have been identified recently. Adherens junction-associated protein 1 (AJAP1, also known as SHREW1) is a membrane protein that is reported to interact with and subsequently sequester β-catenin in the cytosol to inhibit the activation of Wnt/β-catenin signaling (20). AJAP1 is downregulated in several malignancies, including glioma, hepatocellular carcinoma, and gastric cancer (21–23). However, it remains to identify the regulation of AJAP1 in cancer development.

Herein we sought to determine whether TET1 acts a critical role in bladder carcinogenesis and whether the increase of TET1 activity by vitamin C can suppress tumorigenicity. We also exploited gene expression profiling to identify one key downstream target gene AJAP1, whose promoter is hydroxymethylated by TET1. We also examined whether AJAP1 is a critical regulator of TET1-induced tumor suppression and inhibition of Wnt/β-catenin pathway. Our data revealed that the downregulation of TET1 and AJAP1 can predict worse clinical outcomes in UBC patients.

## Materials and Methods

### Cell Lines and Chemicals

Human UBC cell lines (5637, T24, J82, SCaBER, SW780, and UMUC-3) and nonmalignant urothelial cell line (SV-HUC-1) were obtained from Cell Bank of Type Culture Collection, Chinese Academy of Sciences (Shanghai, China). These cell lines were maintained in RPMI 1640 medium supplemented with 10% fetal bovine serum (FBS) and 1% antibiotics (100 U/ml penicillin and 100 μg/ml streptomycin) at 37°C in a humidified incubator containing 5% CO_2_. Vitamin C (L-ascorbic acid), 5-aza-dC, and 3-(4,5-dimethylthiazol-2-yl)-2,5-diphenyltetrazolium bromide (MTT) reagent were purchased from Sigma-Aldrich (St. Louis, MO, USA).

### Construction of Plasmids and Stable Cell Line Establishment

The TET1 cDNA-containing catalytic domain (CD) was subcloned from pCMV3-C-GFPSpark-TET1 plasmid (Cat# HG19726-ACG; Sino Biological, Inc., Beijing, China) into pCDH-3 × FLAG plasmid. TET1-CDmut (H1672Y/H1674A) with two amino acid substitutions in CD regions (enzymatically inactive) was generated from pCDH-3 × FLAG-TET1CD plasmid with Mut Express II Fast Mutagenesis Kit (Cat# C214-01; Vazyme, Nanjing, China). PCR primer for subcloning are listed in Table S1. Two shRNA plasmids targeting TET1 were constructed using the lentiviral pLKO.1 backbone with puromycin resistance. The sequences for TET1-targeting shRNAs were as follows: shTET1-1: 5′-GCAGCTAATGAAGGTCCAGAA-3′; and shTET1-2: 5′-CCCAGAAGATTTAGAATTGAT-3′. Lentiviral particles were produced in 293FT cells co-transfected with the respective plasmid, an envelope plasmid (VSVG) and a packing plasmid (gag-pol). UBC cells were transfected with virus particles, and the infected cells were selected by 1 μg/ml puromycin (Cat# ISY1130; Yeasen, Shanghai, China) for 7 days. Knockdown and overexpression efficiency were determined by RT-PCR and Western blotting.

### Transient Transfections

For siRNA-mediated knockdown, siRNAs were synthesized by GenePharma (Shanghai, China), and transient transfections were performed using Lipofectamine 3000 (Thermo Fisher Scientific) transfection reagent according to the manufacturer's protocol. For functional assays, all siRNA transfections were for at least 24 h in a 50-nM concentration. The sequences were as follows: siAJAP1: 5′-CCACAGAGACUGAGUUCAU-3′; siNC (noncoding control): 5′-UUCUCCGAACGUGUCACGU-3′.

### Immunohistochemistry

Formalin-fixed, paraffin-embedded specimens were from 88 patients diagnosed with UBC at the Department of Urology, Shanghai General Hospital, affiliated with Shanghai Jiaotong University, between 2007 and 2015. The ethics committees of the Shanghai General Hospital approved the protocol. Paraffin sections were deparaffinized in xylene for antigen retrieval, followed by the incubation with anti-TET1 (1:500, Cat# 124207; GeneTex, CA), anti-5 hmC (1:1,000, Cat# 39769; Active Motif, Carlsbad, CA), anti-AJAP1 (1:250, Cat# 223117; Abcam, Cambridge, UK), anti-β-catenin (1:500, Cat# 610153; BD Biosciences, San Jose, CA) antibody at 4°C overnight. On the next day, the 3,3′-diaminobenzidine (DAB) kit (DAB-0031; Maixin Bio, Fujian, China) was applied to visualize the localization of the antigen and counterstain sections with hematoxylin. Antibodies are listed in Table S2. Briefly, staining score of each slide was evaluated through staining intensity (0 = no staining; 1 = weak staining; 2 = moderate staining; 3 = intense staining) and percentage of positive tumor cells (1 = 0–25%; 2 = 25–49%; 3 = 50–75%; 4 = 75–100%). A final score was given by multiplying the staining with the intensity score between 0 and 12. A score of 0–6 signals low expression, whereas 7–12 indicates high expression.

### Immunofluorescence

T24 cells were transfected with siAJAP1 or siNC for 48 h, cells were seeded on glass slides, fixed in 4% paraformaldehyde, and blocked by 1% bovine serum albumin (BSA)/phosphate buffered saline (PBS), followed by permeabilization with 0.1% Triton X-100 for 30 min. Then cells were incubated overnight at 4°C with primary antibodies against with anti-β-catenin antibody (1:400, Cat# 610153; BD Biosciences, San Jose, CA). Alexa 555-conjugated secondary antibody was added for 1 h at room temperature. Finally, cells were counterstained with 4′,6-diamidino-2-phenylindole (DAPI), and images from three replications were captured (Olympus, Tokyo, Japan). Antibodies are listed in Table S2. All experiments were performed three times.

### Western Blotting

Cells were lysed by radioimmunoprecipitation assay (RIPA) buffer containing phosphatase and protease inhibitor cocktail. Twenty micrograms of proteins in the lysates were separated by sodium dodecyl sulfate–polyacrylamide gel electrophoresis (SDS-PAGE) and transferred onto polyvinylidene fluoride (PVDF) membrane (Millipore, Billerica, MA, USA). After blocking with 5% nonfat milk in PBST, the membrane was incubated with primary antibodies overnight at 4°C. Glyceraldehyde-3-phosphate dehydrogenase (GAPDH) was used as a loading control. The membranes were then incubated with secondary antibody respectively. The Western blots were visualized using the enhanced chemiluminescence (ECL) substrate kit (Tanon Science & Technology, Shanghai, China). Antibodies are listed in Table S2. All experimental procedures were repeated at least twice.

### RNA Isolation and Quantitative Reverse Transcription-PCR

Total RNA was extracted using TRIzol reagent (TaKaRa, Dalian, China) and then reverse transcribed into cDNA by Prime-Script RT-PCR kit (TaKaRa, Dalian, China) according to manufacturer's instructions. For AJAP1 detection, 5637 shC and shTET1 cells were seeded in 6-well plates, treated with vehicle or 0.25 mM vitamin C for 72 h, followed by RNA extraction and reversely transcribed into cDNA by the aforementioned method. The expression levels of genes were detected with SYBR Green (high ROX) in an ABI StepOne Real Time PCR instrument (Applied Biosystems, USA). All targets and references were amplified in triplicate. The relative amount of mRNA was normalized by β-actin. Primer sequences for qRT-PCR were listed in Table S1.

### Transwell Invasion Assay

Eight-micrometer transwell filters were used to evaluate the invasive capacity of UBC cells. Mixture of Matrigel and serum-free medium (1:8) was added to the upper chamber and then incubated at 37°C 2 h for gelling. RPMI 1640 culture medium with 10% FBS was added to the bottom well. 1 × 10^5^ cells in 100 μl were then seeded in the upper chamber. After incubation for 13 h (T24 cells) and 18 h (5,637 cells), the UBC cells were fixed with 4% formaldehyde for 15 min and stained with 0.1% crystal violet for 10 min at room temperature. The numbers of invaded cells were counted in three randomly selected fields under a microscope (Leica Microsystems, Wetzlar, Germany). All experiments were performed three times.

### Wound Healing Assay

Briefly, cells were seeded into 6-well plates per well until confluent. The monolayers were scratched with a 100-μl pipette tip and cultured in serum-free medium. Images of the scratch were taken at 0, 12, and 24 h to evaluate the wound closure. All experiments were performed three times.

### Dot Blot Analysis

Genomic DNA was extracted with a QIAamp DNA Mini Kit according to the manufacturer's instructions (Cat# 51304; Qiagen, Germany). The DNA samples were then sonicated and denatured *via* incubation at 95°C for 10 min. Equal amounts of DNA were loaded onto the membranes. After UV cross-linking at 1,200 J/m^2^ and being blocked with 5% nonfat milk for 1 h at room temperature, the membrane was incubated with primary antibodies against 5 hmC (1:10,000, Cat# 39769; Active Motif, Carlsbad, CA) overnight at 4°C. Membranes were incubated with secondary antibodies, and the DNA was detected using ECL substrate kit (Tanon Science & Technology). The membranes were stained with methylene blue (Sangon BioTech, Shanghai, China) to ensure equal loading.

### Cell Proliferation Assay

The cell proliferation assay was performed using MTT reagent (Sigma). For each cell line, 2,000 cells per well were seeded in a 96-well plate. After 12 h, which is identified as 0 time point, 10 μl MTT was added in each well and incubated at 37°C for 4 h. After the MTT solution was removed, 100 μl dimethylsulfoxide (DMSO) was added into each well and incubated at 37°C for 10 min. The detected absorbance is 490 nm. Similar procedure was performed at 24, 48, and 72 h. All proliferative assays were repeated independently for three times.

For cell viability assay, 5,637 shC and shTET1 cells were seeded at 2,000 cells per well in a 96-well plate. Cells were treated with vehicle or 0.25 mM vitamin C. After 72 h, 10 μl MTT was added in each well and incubated at 37°C for 4 h. After the MTT solution was removed, 100 μl DMSO was added into each well and incubated at 37°C for 10 min. The detected absorbance is 490 nm.

### Colony Formation Assay

UBC cells were plated at 500 cells for each well in 6-well plates and treated with or without vitamin C for 14 days. In addition, 5,637 shC and shTET1 cells were plated at 500 cells for each well in 6-well plates and treated with vehicle or 0.25 mM vitamin C for 10 days. Cells were washed by PBS, fixed with formaldehyde, and stained with 0.1% crystal violet at room temperature. Colonies (≥50 cells/colony) were counted in each dish.

### Apoptosis Assay

The apoptosis assay was performed by Annexin V–fluorescein isothiocyanate (FITC)/propidium iodide (PI) double staining kit (Cat# 40302; Yeasen, Shanghai, China), according to the manual's instruction. In brief, T24 cells were plated on 6-well plates at a density of 2 × 10^5^ cells/well to assess apoptosis. After incubation for 48 h treated with or without vitamin C, cells were harvested and resuspended in 100 μl binding buffer containing 10 μl Annexin V–FITC and 5 μl PI. After 15 min incubation at room temperature in the dark, samples were then analyzed by flow cytometry with FACSCalibur (BD Biosciences). Results of apoptotic assay were obtained from three different replications.

### 5-Aza-dC Treatment

The demethylation agent 5-aza-deoxycytidine (5-aza-dC, Cat# A3656; Sigma-Aldrich, St. Louis, MO) was added to the culture medium at the concentration of 10 μM. Cells were harvested, and mRNA levels were analyzed by quantitative real-time RT-PCR 5 days after 5-aza-dC treatment.

### Soft Agar Assay

The potential effect of TET1 or vitamin C on anchorage-independent proliferation in human UBC cells were measured by soft agar assay, as described previously (24). Briefly, 2 × 10^3^ of T24 (Vector), T24 (TET1-CD), or T24 (TET1-CDmut) cells were mixed with 0.35% low-melt agarose (the upper layer) on the bottom layer of 0.7% low-melt agarose containing 0.1 mM vitamin C or not in each well of 6-well plate. Cells were then cultured for 14 days at 37°C, 5% CO_2_, and colonies were then stained with p-iodonitrotetrazolium violet (Sigma-Aldrich) overnight. Colonies were photographed, and the ones with >100 μm in diameter were chosen.

### *In vivo* Xenograft Models

Five-week-old male athymic nude mice nu/nu were purchased from The Model Animal Research Center of Nanjing University (Nanjing, China). A total of 5 × 10^6^ T24 Vector or TET1-CD cells resuspended in 100 μl PBS were subcutaneously (s.c.) injected into one flank of mice. Treatment was initiated when the xenografts reached approximately 150 mm^3^. Vitamin C (4 g/kg) or vehicle (saline) was administered intraperitoneally everyday. The animal protocol was approved by the Institutional Animal Care & Use Committee of Model Animal Research Center of Nanjing University.

### Transcriptome and Bioinformatics Analysis

Transcriptome analysis was performed by GENEWIZ Biotechnology Co. (Suzhou, China). Briefly, total RNAs in control or TET1-CD overexpression cells were extracted using Trizol reagent. Next-generation sequencing library preparations were constructed according to the manufacturer's protocol. Then libraries with different indices were multiplexed and loaded on an Illumina HiSeq instrument (Illumina, San Diego, CA). Differential expression analysis used the DESeq2 Bioconductor package, and differentially expressed genes (DEGs) were identified with a fold change ≥2 and *p* < 0.05. DEGs were then subjected to heatmap and enrichment analysis of GO analysis. The RNAseq data were uploaded with GEO accession No. GSE137646.

### Co-immunoprecipitation

Cells were lysed in CLB buffer (50 mM Tris pH8.0, 100 mM NaCl, 10 mM NaF, 1 mM Na_3_VO_4_, 1% NP-40, 10% Glycerol) supplemented with protease inhibitors (protease inhibitor cocktail, M5293, AbMole BioScience) and phosphatase inhibitors (phosphatase inhibitor cocktail set A, and B, M7528, AbMole BioScience). The protein concentrations of lysates were measured by the BCA protein assay kit (23225, Thermo). The same amounts of whole cell lysates were resolved by SDS-PAGE and immunoblotted with indicated antibodies. For immunoprecipitation, 1,000-μg lysates were incubated with the indicated antibody (1 μg) for 3–4 h at 4°C followed by 1 h incubation with Protein A/G Sepharose beads (Protein A/G plus-agarose, sc-2003; Santa Cruz Biotechnology, Inc., Dallas, TX). Immunoprecipitants were washed five times with N, NaCl; E, EDTA; T, Tris; N, NP-40 (NETN) buffer (20 mM Tris, pH 8.0, 100 mM NaCl, 1 mM EDTA, and 0.5% NP-40) before being resolved by SDS-PAGE and immunoblotted with indicated antibodies.

### Hydroxymethylated DNA Immunoprecipitation

Hydroxymethylated DNA immunoprecipitation (hMeDIP) assay was performed with the hMeDIP kit (Cat# 55010; Active Motif, Carlsbad, CA). Briefly, genomic DNA extracted from cells was sonicated to an average fragment size of 200–500 bp, 10% as input. Then, add reagents and 4 μl of anti-5 hmC in the order list to each IP reaction, rabbit IgG as negative control. After overnight incubation with end-to-end rotation at 4°C, 25 μl of protein G magnetic beads were added and incubated for 2 h at 4°C. After three washes with Buffer C and two wash times with Buffer D, DNA was eluted with Proteinase K, then purified using the Chromatin IP DNA Purification Kit (Cat# 58002; Active Motif) according to the manufacturer's instructions. DNA was analyzed by quantitative real-time PCR. Primer sequences are provided in Table S1. The hMeDIP assay was performed in triplicate.

### Fractionation of Cytosol and Nuclear Protein Lysate

A total of 2 × 10^6^ T24 cells were lysed using Nuclear and Cytoplasmic Protein Extraction Kit (Cat# 20126ES50;Yeasen). Samples were normalized for protein concentration using Pierce BCA Protein Assay. Then, 10 μg of each cytosolic and nuclear extract sample were analyzed by SDS-PAGE and Western blotting using specific antibodies.

### Statistical Analysis

All data were analyzed with GraphPad Prism 6.0 and presented as means ± SD of three independent experiments. Statistical significance was assessed by Student's *t*-test to compare the means of two groups. Pearson correlation test was used to determine the correlation of different gene expressions. Kaplan–Meier survival curves with log-rank test were used to analyze overall survival (OS). ^*^p < 0.05, ^**^p < 0.01, and ^***^p < 0.001 were considered significant.

## Results

### Ten-Eleven Translocation 1 Was Downregulated in Human Urinary Bladder Cancer Tissues, and Its Low Expression Predicts Poor Prognosis in Urinary Bladder Cancer Patients

**Figure 1:**
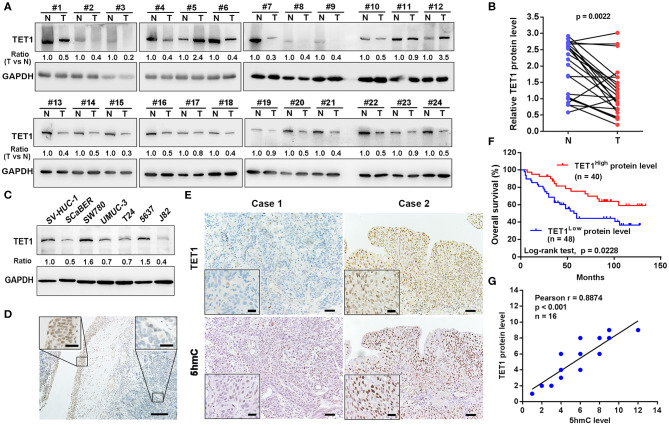
Ten-eleven translocation 1 (TET1) was underexpressed in human urinary bladder cancer (UBC) tissues, and its low expression predicts poor prognosis in UBC patients. **(A)** The expression of TET1 protein in 24 pairs of UBC (T) and matched adjacent normal bladder tissues (N) by Western blotting. **(B)** TET1 protein expression levels by quantitation of densities of protein bands from Western blot in UBC tissues relative to the normal (*n* = 24, *p* = 0.0022) by ImageJ software. **(C)** Endogenous expression of TET1 in various human UBC cell lines and a nonmalignant normal urothelial cell line by Western blotting. **(D)** Immunohistochemical (IHC) staining of TET1 expression in a section containing cancerous (right insect) and its adjacent normal urothelial cells (left insect). Scale bar, 100 and 20 μm (inset). **(E)** The representative IHC images of TET1 and 5-hydroxymethylcytosine (5 hmC) expression in UBC tissues. **(F)** Kaplan–Meier analysis of the correlation between TET1 expression and overall survival (OS) in UBC patients through IHC score (*n* = 88). **(G)** The correlation of TET1 and 5 hmC expression in UBC tissues were analyzed by Pearson correlation coefficient analysis. The correlation coefficient and *p*-values are shown. Scale bar, 50 and 20 μm (inset).

We first examined the expression levels of TET1 in 24 pairs of UBC tissues and their corresponding adjacent normal bladder tissues. As shown in Figures 1A,B, TET1 was reduced in 75.0% UBC tissues at protein levels (*n* = 24). We next examined a panel of immortalized normal urothelial and UBC cell lines. As shown in Figure 1C, TET1 protein expression level was lower in most UBC cell lines than in a nonmalignant normal urothelial cell line SV-HUC-1. Consistently, we observed that TET1 remarkably expresses in the nuclei of normal urothelial cells, but its expression levels reduce in UBC cells on the same section of human UBC patient specimen by immunohistochemical (IHC) staining (Figure 1D). Representative images of varying staining intensities of TET1 in a cohort of 88 UBC samples are also shown in Figure S1 and Figure 1E. We found that TET1 protein level is significantly associated with T stage (*p* = 0.002) and tumor grade (*p* < 0.001; Table 1). IHC data also demonstrated that compared with the patients with high TET1 expression levels, patients with low expression of TET1 showed poor survival outcomes (*p* = 0.023; Figure 1F). Furthermore, there was a significant correlation between TET1 and 5 hmC levels, suggesting that TET1 expression is positively associated with its enzymatic activity in UBC specimens (*n* = 16, R = 0.8874, *p* < 0.001; Figures 1E,G). These data suggest that the low level of TET1 could be related to cancer progression and poor survival of UBC patients.

**Table 1:** The association between ten-eleven translocation 1 (TET1) protein levels and clinicopathological features of urinary bladder cancer (UBC) patients.

**Characteristics**	**Number**	**Expression of TET1**		***p*-value**
		**High (*n*, %)**	**Low (*n*, %)**	
Gender				0.88
Male	72	33 (45.8%)	39 (54.2%)	
Female	16	7 (43.8%)	9 (56.2%)	
Age				0.968
≥65	53	24 (45.3%)	29 (54.7%)	
<65	35	16 (45.7%)	19 (54.3%)	
T stage				**0.002**
Ta-1	50	30 (60.0%)	20 (40.0%)	
T2-4	38	10 (26.3%)	28 (73.7%)	
Tumor grade				**<0.001**
Low	33	24 (72.8%)	9 (27.2%)	
High	55	16 (29.1%)	39 (70.8%)	
N stage				0.332
N0	77	37 (48.1%)	40 (51.9%)	
≥N1	11	3 (27.3%)	8 (72.7%)	

*Numbers in bold indicate p value with statistical difference*.

### Knockdown of Ten-Eleven Translocation 1 Facilitates Urinary Bladder Cancer Cell Proliferation and Invasion

**Figure 2:**
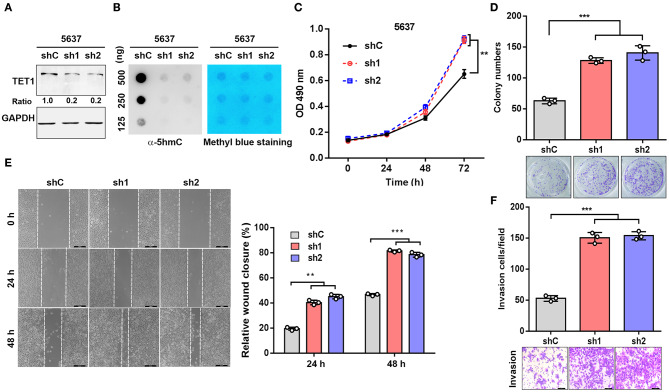
Knockdown of ten-eleven translocation 1 (TET1) facilitates urinary bladder cancer (UBC) cell proliferation and invasion. **(A,B)** The efficiency of knockdown of TET1 **(A)** and 5-hydroxymethylcytosine (5 hmC) level **(B)** in 5,637 cell line were confirmed by Western blotting and dot blot, respectively. **(C–F)** Effects of TET1 knockdown on cell proliferation **(C)**, colony formation **(D)**, migration **(E)**, and invasion **(F)** of UBC cells. Scale bar, 200 μm **(E)** and 100 μm **(F)** **p < 0.01, ***p < 0.001.

To explore the role of TET1 in UBC development, 5,637 cells with high TET1 expression were infected with lentiviruses expressing short hairpin RNA targeting TET1 (sh1 and sh2) and control shRNA (shC). TET1 expression and 5 hmC levels were decreased in TET-knockdown cells compared to shC cells (Figures 2A,B). The depletion of TET1 in 5637 cells significantly stimulated cell proliferation, as determined by MTT analysis and colony formation assay (Figures 2C,D). Furthermore, wound-healing and transwell invasion assays demonstrated that TET1 deficiency also significantly promoted migration and invasion capacities of UBC cells (Figures 2E,F). Meanwhile, we also knocked down TET1 in nonmalignant urothelial cell line SV-HUC-1 (Figures S2A,B), with the reduction of 5 hmC levels (Figure S2C). TET1 deficiency also promotes cell proliferation and invasiveness of SV-HUC-1 cells (Figures S2D,E). In summary, these data provided the evidence that loss of TET1 could induce cell proliferation and invasion of UBC and nonmalignant urothelial cells.

### Ectopic Expression of Ten-Eleven Translocation 1-CD and Vitamin C Treatment Reduce Urinary Bladder Cancer Cell Proliferation and Invasion, Dependent on Its Catalytic Domain

**Figure 3:**
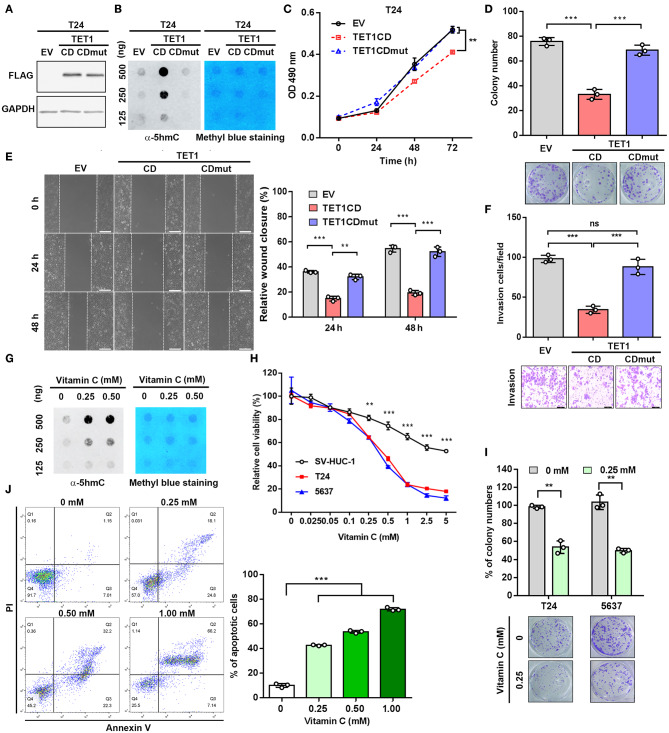
Ectopic expression of ten-eleven translocation 1 (TET1)-CD and vitamin C treatment reduce urinary bladder cancer (UBC) cell proliferation and invasion, dependent on its catalytic domain. **(A)** Confirmation of overexpression of TET1-CD by Western blotting. **(B)** Dot blot assay of 5-hydroxymethylcytosine (5 hmC) level in TET1-CD and TET1-CDmut overexpression T24 cells, as well as empty vector (EV) control cells with DNA concentration gradients. **(C–F)** Effects of TET1 overexpression on cell viability **(C)**, colony formation **(D)**, migration **(E)**, and invasion **(F)** *in vitro*. **(G)** Detection of 5 hmC levels of T24 cell treated with various doses of vitamin C by dot blot assay. **(H)** 3-(4,5-Dimethylthiazol-2-yl)-2,5-diphenyltetrazolium bromide (MTT) assay of T24, 5,637 UBC and nonmalignant SV-HUC-1 urothelial cell viabilities at various concentrations with vitamin C treatment for 48 h. **(I)** Colony formation assay for T24 and 5,637 cells at various concentrations of vitamin C. **(J)** Apoptosis assay of T24 cells treated with various concentrations with vitamin C for 48 h. Scale bar, 200 μm **(E)** and 100 μm **(F)**. **p < 0.01, ***p < 0.001.

To further investigate whether the enzymatic activity of TET1 protein is disposable for its tumor-suppressive effects on UBC cells, we stably ectopically expressed TET1 catalytic domain (TET1-CD) and its enzymatically inactive mutant (TET-CDmut) in T24 cells, which express low level of endogenous TET1 protein (Figure 3A). Consistently, TET1-CD overexpression cells showed increased 5 hmC level compared with empty-vector (EV) transfectants, whereas TET1-CDmut transfectants possessed a low 5 hmC level comparable to EV control cells (Figure 3B). MTT and colony formation assay demonstrated that TET1-CD, but not its mutant form (TET1-CDmut) overexpression cells, significantly suppressed cell viability on the third day (*p* < 0.01; Figure 3C) and decreased colony formation (*p* < 0.001; Figure 3D) by approximately 50% reduction compared with EV control cells. Transwell invasion and wound-healing assays revealed that the forced expression of TET1-CD significantly inhibited migration and invasion of T24 cells (*p* < 0.001; Figures 3E,F). But TET1-CDmut transfectants did not show any obvious inhibitory effects on cell proliferation, colony formation, migration, and invasion compared to EV control cells (Figures 3C–F). These data demonstrated that TET1 restrained UBC cell aggressiveness dependent on its catalytic activity.

Vitamin C is a cofactor for Fe (II)-2-oxoglutarate-dependent dioxygenases, such as TET1 (25). Recently, it was reported that vitamin C could alleviate leukemia malignant progression *via* enhancing TET-mediated 5 hmC content (26). Therefore, we argued whether the increase of TET1 activity by vitamin C can mimic the anticancer effects of TET1-CD overexpression. Dot blot assay confirmed the increased 5 hmC levels in UBC cell with vitamin C treatment in a dose-dependent manner (Figure 3G). MTT assay further demonstrated that vitamin C suppressed cell proliferation of UBC cells more strikingly than SV-HUC-1 cells after 48 h treatment (Figure 3H). Colony formation assay also showed the markedly cytotoxic effects of vitamin C on T24 and 5,637 cells (Figure 3I). Besides that, when UBC cells were treated with high-dose vitamin C, apoptotic rates increased in a concentration-dependent manner (Figure 3J). To test whether TET1 expression is required for vitamin C, we treated 5,637 shC and shTET1 cells with vitamin C. As shown in Figures S3A–C, vitamin C suppressed 5,637 cell viability and colony formation capacity, such effects were not significantly different in TET1 deficient cells. Taken together, the restoration of global 5 hmC level in UBC cells by both the forced expression of TET1-CD and vitamin C treatment can significantly suppress UBC cell proliferation and survival.

### Ten-Eleven Translocation 1 or Vitamin C Treatment Effectively Suppresses Urinary Bladder Cancer Tumorigenicity

**Figure 4:**
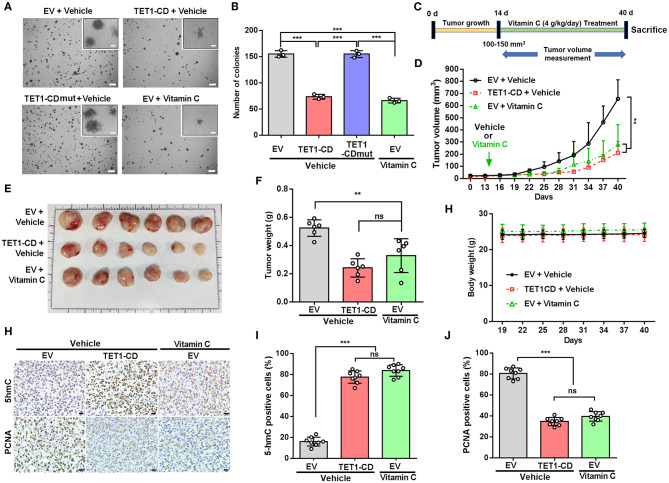
Ten-eleven translocation 1 (TET1) or vitamin C treatment effectively suppresses urinary bladder cancer (UBC) tumorigenicity. **(A,B)** Representative images **(A)** and quantification of the colony numbers **(B)** of TET-CD, TET1-CDmut overexpression T24, and vitamin C-treated T24 cells by soft agar assay. Scale bar, 1 mm. Inset, scale bar, 500 μm. **(C)** Experimental design for vitamin C treatment in xenograft mouse model (*n* = 6 per experimental cohort). **(D)** Tumor growth curves of T24 xenografts with vector-transfected or TET1-CD overexpression treated with or without vitamin C. Tumor volume is shown as the mean ± SD (*n* = 6). **(E,F)** Images **(E)** and weight **(F)** of xenograft tissues after injection with vector-transfected cells and TET1-CD-transfected cells treated with or without vitamin C at the end point (*n* = 6). **(G)** The body weight of mice with varying treatment (*n* = 6). **(H–J)** Immunohistochemical (IHC) staining of 5-hydroxymethylcytosine (5 hmC) and proliferating cell nuclear antigen (PCNA) in T24 xenografts of different group. Scale bar, 20 μm. Eight fields from each specimen were randomly chosen and cells with positive nuclear staining were counted **(I,J)**. **p < 0.01, ***p < 0.001, ns, not significant.

To further elucidate the role of TET1 or vitamin C in bladder carcinogenesis, soft agar assays were performed to evaluate the anchorage-independent growth of UBC T24 cells. The results showed that TET1-CD overexpression and vitamin C treatment resulted in the reduced colony number of UBC cells, respectively (*p* < 0.001; Figures 4A,B). To figure out the role of TET1 in bladder carcinogenesis *in vivo*, T24 empty vector or TET1-CD overexpressed cells were subcutaneously (s.c.) inoculated into the flanks of nude mice (Figure 4C). Our data showed that the xenografts in groups of TET1-CD stable overexpression cells or vitamin C treatment were much smaller than those in the EV control group (*p* < 0.01; Figure 4D and Figure S4A). At the endpoint, the average weights of xenografts in TET1-CD overexpression or vitamin C-treated group were significantly lighter than those of control group (*p* < 0.01; Figures 4E,F). Meanwhile, vitamin C did not have an obvious impact on body weights of mice throughout the treatment period, suggesting that the dosage of vitamin C we used is relatively safe (Figure 4G). Consistently, increased 5 hmC levels in both TET1-CD xenografts and vitamin C-treated xenografts were confirmed by IHC staining (Figures 4H,I). Furthermore, in comparison with the EV control group, the percentages of proliferating UBC cells [proliferating cell nuclear antigen (PCNA)-positive] significantly decreased in TET1-CD overexpression and vitamin C-treated xenografts, respectively (Figures 4H,J). These data indicate that TET1 plays an essential role in UBC suppression, whereas vitamin C can increase 5 hmC levels and impair bladder carcinogenesis.

### Adherens Junction-Associated Protein 1 Is Critical for Ten-Eleven Translocation 1-Mediated Inhibition of Tumor Progression in Urinary Bladder Cancer

**Figure 5:**
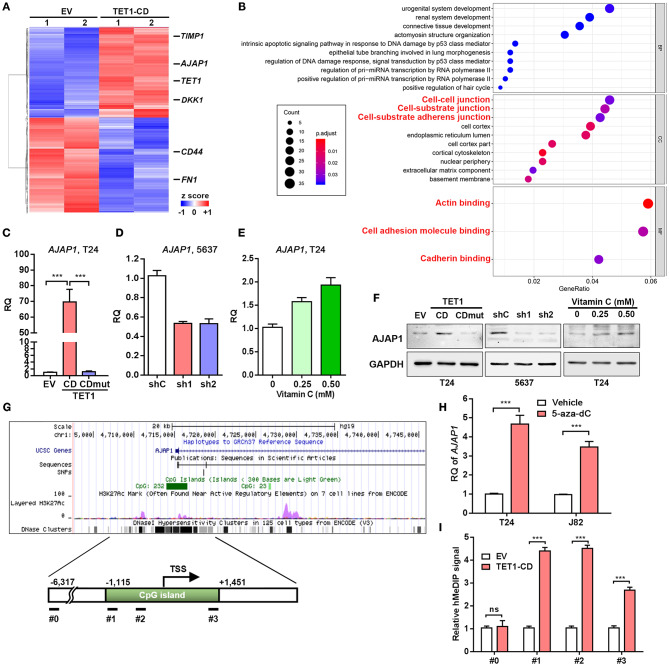
Adherens junction-associated protein 1 (AJAP1) is critical for ten-eleven translocation 1 (TET1)-mediated inhibition of tumor progression in urinary bladder cancer (UBC). **(A)** Heatmap of most differentially expressed genes (DEGs) of vector and TET1-CD-transfected T24 cells. **(B)** Gene Ontology (GO) term analysis from DEGs. **(C–E)** The expression levels of AJAP1 in TET1-CD-transfected T24 cells **(C)** and TET1 knockdown 5,637 cells **(D)** and vitamin C-treated T24 cells **(E)** were examined by qRT-PCR, respectively. **(F)** The expression of AJAP1 in TET1-CD-transfected T24 cells and TET1 knockdown 5,637 cells and vitamin C-treated T24 cells by Western blotting. **(G)** Schematic of CpG island on the AJAP1 transcription initiation site (TSS) using UCSC website. **(H)** The elevated level of AJAP1 expression after 5-day treatment of 5-aza-deoxycytidine (5-aza-dC; 10 μm) in T24 and J82 cells by qRT-PCR. **(I)** Enrichment of 5-hydroxymethylcytosine (5 hmC) in the promoter of AJAP1 was detected by hMeDIP-qPCR in empty vector (EV) and TET1-CD-transfected T24 cells. ***p < 0.001.

To uncover the mechanism involved in the TET1-mediated UBC suppression, RNAseq analysis of DEGs was performed (Figure 5A). While TET1 was ectopically expressed by ~35-fold in the TET1-CD overexpression T24 cells, several previously identified TET1 target genes, such as TIMP3 and DKK1 in other cancer types (13, 14), were also induced at 2.1- and 1.8-fold compared to the control cells (EV), respectively. These data indicate the reliability of our expression profiling data. Next, we performed gene ontology (GO) analysis, which revealed that genes involved in cell–cell junction and cell–substrate AJ are enriched in TET1-CD overexpression cells (Figure 5B). Since AJs have been reported to play a critical role in carcinogenesis (27), we validated the RNAseq data and found AJAP1, a novel molecule of AJ, showed the upregulation by ~70-fold in TET1-CD transfectants at the mRNA level, but not in the enzymatically inactive mutant TET1-CDmut transfectants (Figure 5C). The expression level of AJAP1, which was detected on the cell membrane by immunofluorescence, was shown in Figure S6. Its upregulation by TET1-CD at protein level was also confirmed by Western blotting (Figure 5F). Congruently, the depletion of TET1 in 5,637 cells strikingly reduced AJAP1 expression at mRNA and protein levels (Figures 5D,F). When we treated T24 cells with vitamin C to increase 5 hmC level, AJAP1 was also upregulated in a dose-dependent manner (Figures 5E,F). Similarly, vitamin C increased AJAP1 expression in 5,637 cells, such effects were not significantly different in TET1 deficient cells. (Figure S3D). In xenograft tissues, we also observed that overexpression of TET1-CD and vitamin C treatment induced AJAP1 expression at protein level (Figure S4B). Immunofluorescence analysis also confirmed that overexpression of TET1-CD induces the expression level of AJAP1 on the cell membrane of T24 cells (Figure S5). We observed that the depletion of TET1 in nonmalignant urothelial SV-HUC-1 cells also reduces AJAP1 at mRNA level (Figures S2A,F), suggesting that such regulation of AJAP1 by TET1 is not cell line dependent.

Interestingly, in an independent GEO dataset (NCBI/GEO/GSE13507), we found that AJAP1 was downregulated in UBC tissues, particularly in muscle-invasive UBC specimens than in superficial UBC specimens (Figure S5A). Moreover, AJAP1 level was negatively correlated with T stage and grade (Figures S5B–D). Notably, patients with low AJAP1 mRNA levels had shorter overall survival time (Figure S5E), suggesting that AJAP1 level may serve as a prognostic marker in UBC patients. Hence, we focused on this AJAP1 gene for further study.

Since AJAP1 is regulated by TET1 which requires its enzymatic activity, we argued TET1 may regulate through its hydroxymethylation on the promoter of AJAP1 gene. Hence, when using UCSC website (http://genome.ucsc.edu/, human CRHg37/hg19), we found AJAP1 gene harbors a CpG island around the transcription start site (TSS; Figure 5G). To confirm AJAP1 was repressed by DNA methylation, we treated two UBC cells (T24 and J82) with the DNA methylation inhibitor 5-aza-dC for 5 days. As shown in Figure 5H, 5-aza-dC treatment significantly increased AJAP1 expression in both cell lines, indicating that AJAP1 is regulated by promoter methylation. Next, we performed hMeDIP assay and proved that ectopic expression of TET1-CD increases the level of 5 hmC in the promoter of AJAP1 gene in T24 cells indicating that TET1 upregulated AJAP1 expression through promoter hydromethylation (Figure 5I). These data suggested that TET1 can promote demethylation of AJAP1 in UBC cells.

### Ten-Eleven Translocation 1 Inhibited Urinary Bladder Cancer Progression by Regulating β-Catenin Through Mediating Adherens Junction-Associated Protein 1 Expression

**Figure 6:**
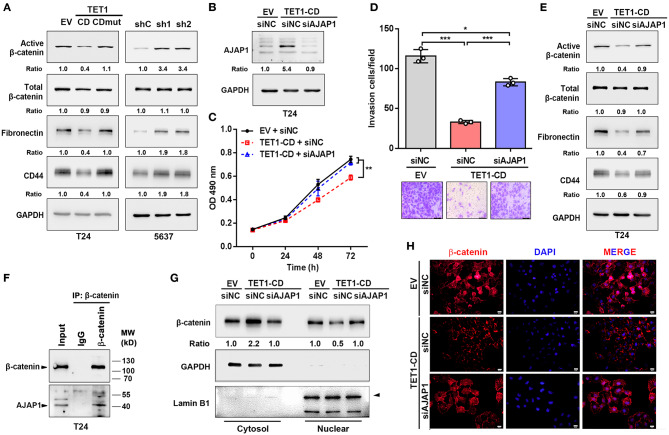
Ten-eleven translocation 1 (TET1) inhibited urinary bladder cancer (UBC) progression by regulating β-catenin through mediating adherens junction-associated protein 1 (AJAP1) expression. **(A)** Western blot analysis showed the expression of β-catenin and the downstream targets (fibronectin and CD44) in empty vector (EV), TET1-CD, TET1-CD-mut-transfected T24 cells and shC, shTET1-transfected 5,637 cells (sh1 and sh2). **(B)** Validation of AJAP1 protein levels of groups after transfected with the siRNA to AJAP1 by Western blotting. **(C,D)** Cell viability and invasion ability of three groups of T24 cells were detected by 3-(4,5-dimethylthiazol-2-yl)-2,5-diphenyltetrazolium bromide (MTT) assay **(C)** and transwell invasion assay **(D)**. **(E)** The levels of β-catenin and the downstream targets of β-catenin were detected with Western blotting in TET1-CD overexpression T24 cells transiently transfected with siNC and siAJAP1. **(F)** Co-IP assays showed that endogenous protein of AJAP1 interacted with β-catenin in T24 cells. **(G,H)** Localization of β-catenin in TET1-CD overexpression T24 cells transiently transfected with siNC and siAJAP1, which were detected with Western blotting **(G)** on the fractionation of cytosol and nuclear compartments and immunofluorescence **(H)** with β-catenin antibodies (red), followed by the counterstaining with 4′,6-diamidino-2-phenylindole (DAPI) (blue). Scale bar, 20 μm. *p < 0.05, **p < 0.01, ***p < 0.001.

It was previously reported that AJAP1 loss can enhance β-catenin translocation into the nucleus to transactivate its downstream target gene (28). Once looking up the DEGs list, we did find several Wnt/β-catenin target genes, such as CD44 and fibronectin (Fn1), were downregulated in TET1-CD transfectants (Figure 5A). Therefore, we hypothesize that TET1 deficiency in UBC cells activates Wnt/β-catenin signaling mediated by AJAP1 loss. To test it, we first examined the influence of TET1 on β-catenin signaling. In T24 cells, overexpression of TET1-CD, but not its mutant form, decreased the levels of active β-catenin and its downstream targets (CD44 and fibronectin) by Western blotting (Figure 6A). Consistently, once we depleted TET1 in 5,637 cells, the levels of both active β-catenin and its downstream targets increased, with no obvious changes in total β-catenin levels (Figure 6A). Next, we explored whether AJAP1 mediated the function of TET1 in UBC progression. Three groups of T24 cells were set up: empty vector (EV), TET1-CD overexpression, and TET1-CD overexpression with AJAP1 knockdown (TET1-CD/siAJAP1; Figure 6B). Compared to the TET1-CD group, the knockdown of AJAP1 in TET1-CD cells significantly enhanced the proliferation rate and partially rescued TET1-CD overexpression-induced loss of cell invasiveness (Figures 6C,D).

When we depleted AJAP1 in TET1-CD overexpressed UBC cells, active β-catenin and its downstream target gene expression were increased compared with TET1-CD group (Figure 6E). Furthermore, co-immunoprecipitation (co-IP) assays confirmed that the endogenous AJAP1 interacted with β-catenin in T24 cells (Figure 6F). Since AJAP1 localizes on the cell membrane, the interaction of AJAP1 and β-catenin may sequester β-catenin in the cytosol. To confirm it, we fractionated cytosol and nucleus compartment and Western blotting data indicated that TET1-CD overexpression promoted the cytosolic localization of β-catenin, with its concomitantly reduced expression in the nuclear compartment. However, such changes of β-catenin localization were reversed by the depletion of AJAP1 (Figure 6G). In line with this, immunofluorescence staining confirmed that the reduced nuclear localization of β-catenin intensity in TET1 overexpression cells, while AJAP1 silencing enhanced the nuclear translocation of β-catenin (Figure 6H). These data suggest a critical role of AJAP1 in TET1 regulating β-catenin signaling in UBC development.

### The Downregulation of Ten-Eleven Translocation 1/Adherens Junction-Associated Protein 1 Axis Predicts Worse Clinical Outcomes

**Figure 7:**
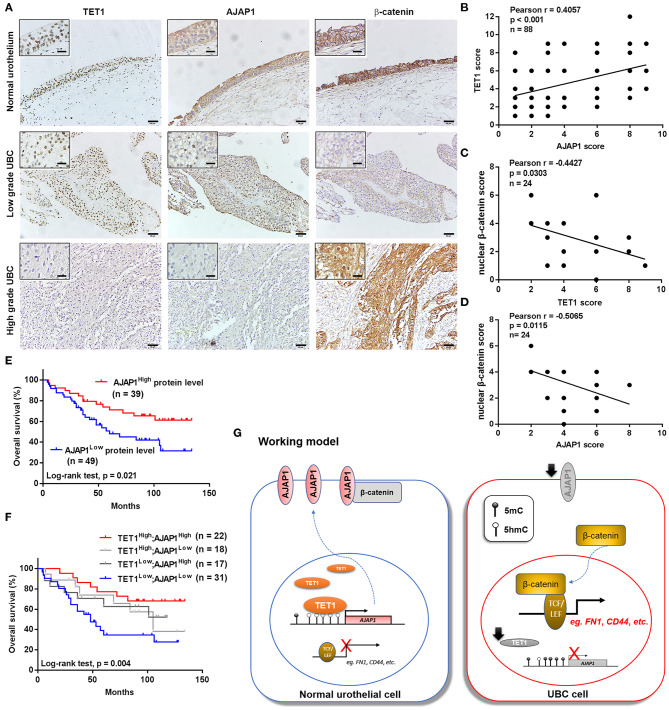
The downregulation of ten-eleven translocation 1 (TET1)/adherens junction-associated protein 1 (AJAP1) axis predicts worse clinical outcomes. **(A)** Representative images of the localization and expression of AJAP1, β-catenin, and TET1 in normal urothelium and urinary bladder cancer (UBC) tissues were presented by immunohistochemical (IHC) staining. Scale bar, 50 and 20 μm (inset). **(B)** The relationship between TET1 and AJAP1 protein expression was determined by Pearson correlation analysis. **(C,D)** The correlation of nuclear expression of β-catenin with TET1 **(C)** and AJAP1 **(D)** in UBC specimens were determined by Pearson correlation analysis. **(E)** Kaplan–Meier survival curve of UBC patients with high AJAP1 and low AJAP1 protein level. **(F)** The correlation between the combined expression of TET1 and AJAP1 with the overall survival of UBC patients were determined by Kaplan–Meier analysis. **(G)** A working model of TET1/AJAP1/β-catenin axis in UBC progression.

To further investigate the clinical relevance of TET1/AJAP1 axis in UBC samples, we interrogated the same cohort of 88 UBC patients and observed that AJAP1 was mainly expressed on the membranes and cytosols of nonmalignant urothelial cells, while its expression was reduced in high-grade UBC samples (Figure 7A). We found that AJAP1 expression was positively associated with TET1 in UBC specimens (*n* = 88, *p* < 0.001; Figure 7B). Both AJAP1 and β-catenin tended to present on the membrane in TET1 overexpressed UBC samples, while AJAP1 level was concomitantly reduced with the enhanced nuclear expression of β-catenin in UBC specimens with low TET1 expression (*n* = 24; Figures 7B–D). We found that AJAP1 protein level was significantly associated with T stage (*p* = 0.003) and tumor grade (*p* = 0.017; Table 2). Furthermore, patients with high AJAP1 level indicated better overall survival than those with low AJAP1 level (*p* = 0.021; Figure 7E). Notably, we found that patients with low expression levels of both TET1 and AJAP1 had worse clinical outcomes, while those with high expression levels of both TET1 and AJAP1 had better prognosis (Figure 7F). Then a Cox regression model was used to analyze the effects of TET1 and AJAP1 expression and other clinicopathological features on the prognosis of patients with UBC. Univariate analysis showed that T stage, grade, TET1 expression, AJAP1 expression, and combined with TET1 and AJAP1 expression were closely related to patient prognosis (Table 3). Multivariate Cox regression analysis showed that co-expression of TET1/AJAP1 was an independent predictor for UBC (Table 3). Taken together, in normal urothelial cells, TET1 can sequester β-catenin on the membrane to prevent its nuclear localization by maintaining the demethylation of AJAP1 promoter, while the downregulation of TET1 in UBC cells fails to maintain AJAP1 expression, thereby leading to the translocation of β-catenin in the nucleus to coactivate TCF transcription activity (Figure 7G).

**Table 2:** The association between adherens junction-associated protein 1 (AJAP1) protein levels and clinicopathological features of urinary bladder cancer (UBC) patients.

**Characteristics**	**Number**	**Expression of AJAP1**		***p*-value**
		**High (*n*, %)**	**Low (*n*, %)**	
Gender				0.106
Male	72	29 (40.3%)	43 (59.7%)	
Female	16	10 (62.5%)	6 (37.5%)	
Age				0.514
≥65	53	22 (41.5%)	31 (58.5%)	
<65	35	17 (48.6%)	18 (51.4%)	
T stage				**0.003**
Ta-1	50	29 (58.0%)	21 (42.0%)	
T2-4	38	10 (26.3%)	28 (73.7%)	
Tumor grade				**0.017**
Low	33	20 (60.6%)	13 (39.4%)	
High	55	19 (34.5%)	36 (65.5%)	
N stage				0.295
N0	77	37 (48.1%)	40 (51.9%)	
≥ N1	11	3 (27.3%)	8 (72.7%)	

*Numbers in bold indicate p value with statistical difference*.

**Table 3:** Univariate and multivariate analysis of clinicopathological features and survival time of patients with urinary bladder cancer (UBC).

**Variables**	**Univariate analysis**		**Multivariate analysis**	
	**HR (95% CI)**	***p*-value**	**HR (95% CI)**	***p*-value**
Age (year)	1.817 (0.864–3.822)	0.115	1,421 (0.660–3.061)	0.370
(<60 vs. ≥60)				
Gender	0.941 (0.448–1.975)	0.872	0.649 (0.289–1.457)	0.295
(female vs. male)				
T stage	2.081 (1.114–3.891)	**0.022**	1.285 (0.643–2.570)	0.477
(Ta-1 vs. T2-4)				
Grade	2.830 (1.380–5.506)	**0.005**	2.020 (0.866–4.710)	0.104
(low vs. high)				
TET1 expression	0.477 (0.249–0.913)	**0.025**	2.077(0.793–5.440)	0.137
(low vs. high)				
AJAP1 expression	0.472 (0.246–0.905)	**0.028**	1.168(0.448–3.046)	0.750
(low vs. high)				
TET1 and AJAP1 expression	0.476 (0.309–0.735)	**0.001**	0.390 (0.182–0.839)	**0.016**
(low/low vs. others vs. high/high)				

*HR, hazard ratio; CI, confidence interval. Numbers in bold indicate p value with statistical difference*.

## Discussion

Abnormalities in DNA methylation status are associated with tumor progression in cancer patients. TET-mediated DNA demethylation was widely reported in differentiation, pluripotency, and cancers (29–31). However, few reports have elucidated its role in UBC, which is one of the most common cancers in the urinary tract. Herein, we found TET1 was expressed in normal bladder urothelium and downregulated or even lost in tumor tissues. The expression of TET1 is sufficient and necessary to restrain the aggressiveness of UBC, which is dependent on its enzymatic activity. The downregulation of TET1 and its direct target gene AJAP1 promotes cancer progression via activation of β-catenin signaling. Finally, we found that UBC patients with both low expression levels of TET1 and AJAP1 had poor clinical outcome. Taken together, these data suggest that TET1 may function as a tumor suppressor gene in UBC cells.

Albeit there are few reports on its genetic mutations, TET1 is frequently downregulated in various solid tumors, including colon and gastric cancer, with the concomitant reduction of global 5 hmC levels (11, 12). However, recent emerging evidences demonstrated its oncogenic role in some cancer types, such as ovarian cancer and triple-negative breast cancer. In ovarian cancer, overexpression of TET1 increases chemoresistance partially through promoting epithelial-to-mesenchymal transition mediated by the induction of vimentin level (32). Interestingly, TET1 expression can be induced under hypoxic microenvironment in multiple cancer cells. The induction of TET1 protein coactivates HIF1α and HIF2α to enhance their transcriptional activity independent of its enzymatic activity, which leads to EMT and cancer cell invasion (33). In a word, the diversified role of TET1 seems to function in a cancer type-dependent or cell content-dependent manner.

In recent years, we have demonstrated the accumulation of β-catenin in clinical UBC tissues, suggesting the significance of β-catenin pathway in UBC progression and metastasis (34). Notably, constitutive activation of β-catenin in basal cells from urothelium initiates tumor formation in mice, which resembles human low-grade papillary urothelial carcinoma (35). Thus, targeting the Wnt/β-catenin signaling might be an effective way to suppress UBC cells. In our study, we found that TET1 inhibited the expression of active β-catenin and its downstream target genes such as CD44 and fibronectin, which requires its catalytic activity. These data demonstrated that TET1 suppressed UBC cell growth by regulating the β-catenin signaling pathway.

Recent studies revealed that TET1 could induce the demethylation on the promoters of a few Wnt antagonists and restore their expressions in nasopharyngeal carcinoma, finally decreasing the expression of nuclear β-catenin (11). To determine how TET1 regulated the downstream pathway in UBC, our RNA-seq and subsequent qPCR analysis obtained from TET1-CD-transfected T24 cell showed a remarkable and significant upregulation of AJAP1, a novel molecule of AJ, which was recently reported to interact with β-catenin and inhibit its nuclear translocation (20). In a GEO dataset, we found AJAP1 was downregulated in UBC tissues and its expression level was negatively correlated with overall survival in UBC patients, suggesting a putative tumor-suppressive role of AJAP1 in bladder carcinogenesis. Besides, AJAP1 transcription was shown to be regulated by promoter hypermethylation (36), suggesting potential correlation of AJAP1 and DNA methylation or demethylation. Herein, our data clearly demonstrated that TET1 indeed significantly restored the expression of AJAP1 through its binding to the CpG islands of AJAP1 and altering the methylation status of this gene. Subsequent AJAP1 depletion in TET1-CD overexpression cells, to a large extent, restored β-catenin nuclear translocation, expression of active β-catenin, and its downstream genes like CD44 and fibronectin. Inhibition of cell proliferation and invasion by TET1 was also abrogated, to some extent, by AJAP1 depletion. However, the effect of TET1 was not fully rescued by AJAP1 depletion, indicating other mechanisms involved in tumor suppression of TET1.

The antitumor effects of vitamin C have been reported in several cancers (37, 38). As a cofactor, vitamin C can strengthen the activity of Fe (II)-2-oxoglutarate dioxygenases, including TET1, leading to DNA demethylation. The suppressive effects of vitamin C caused by the demethylation through activating TET has been reported in several studies. In melanoma, vitamin C could restore 5 hmC levels and rebuild the transcriptome (38). In leukemia, vitamin C has been confirmed to suppress leukemogenesis by promoting TET activity (26). Besides this, vitamin C could also enhance the chemosensitivity and even reduce the toxicity of chemotherapy such as colorectal cancer, ovarian cancer, and hepatocellular carcinoma (39, 40). In UBC, consistent with the above results, we found vitamin C could restore 5 hmC level and inhibit cancer progression *in vitro* and *in vivo*.

In conclusion, our study demonstrated for the first time that TET1 may function as a tumor suppressor gene in UBC and AJAP1 plays a crucial role in TET1 suppression of bladder tumor growth through modulation of the Wnt/β-catenin signaling, affecting its downstream target genes. Furthermore, vitamin C appears to restore 5 hmC level and delay tumor growth. Overall, these data demonstrate the functional role of TET1 loss in UBC progression and provide with an alternative therapeutic target. The underlying mechanism of the inhibitory effect of TET1 on UBCs requires additional study.

## Data Availability Statement

All data are available within the article/Supplementary Material or available from the authors upon request.

## Ethics Statement

The animal protocol was approved by the Institutional Animal Care & Use Committee of Model Animal Research Center of Nanjing University. The studies involving human participants were reviewed and approved by the Shanghai General Hospital. The patients/participants provided their written informed consent to participate in this study.

## Author Contributions

BS, RH, and JY designed the experiments, supervised the progress throughout this study, and revised the manuscript. YY and ZH carried out the experiments and wrote the manuscript. ZZ analyzed the data. YC and ML participated in the experiments. All authors reviewed and approved the final manuscript.

## Conflict of Interest

The authors declare that the research was conducted in the absence of any commercial or financial relationships that could be construed as a potential conflict of interest.
