# Supplementary material for: Downregulation of TET1 Promotes Bladder Cancer Cell Proliferation and Invasion by Reducing DNA Hydroxymethylation of AJAP1
Source: Front Oncol. 2020 May 21;10:667. doi: 10.3389/fonc.2020.00667 (PMC7253684; doi:10.3389/fonc.2020.00667)
Supplement: Supplementary file 1 [file Data_Sheet_1.docx]

Supplementary Material


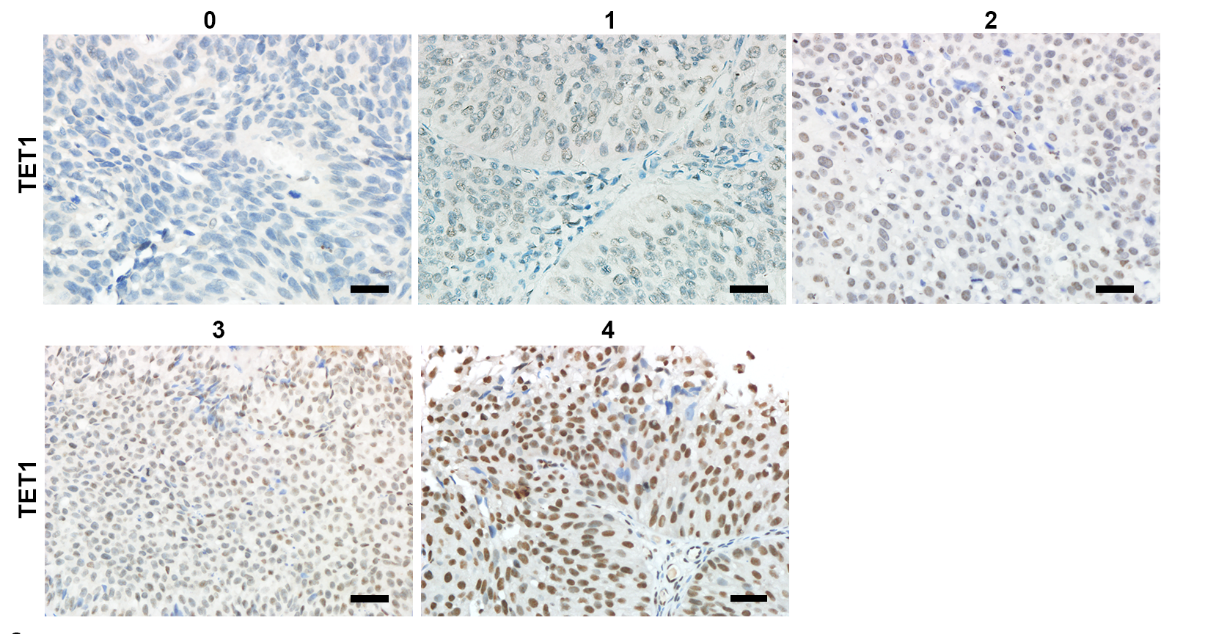


**Figure S1|** Representative images of various staining intensities of TET1 in UBC specimens by IHC staining. Scale bar = 20 μm.


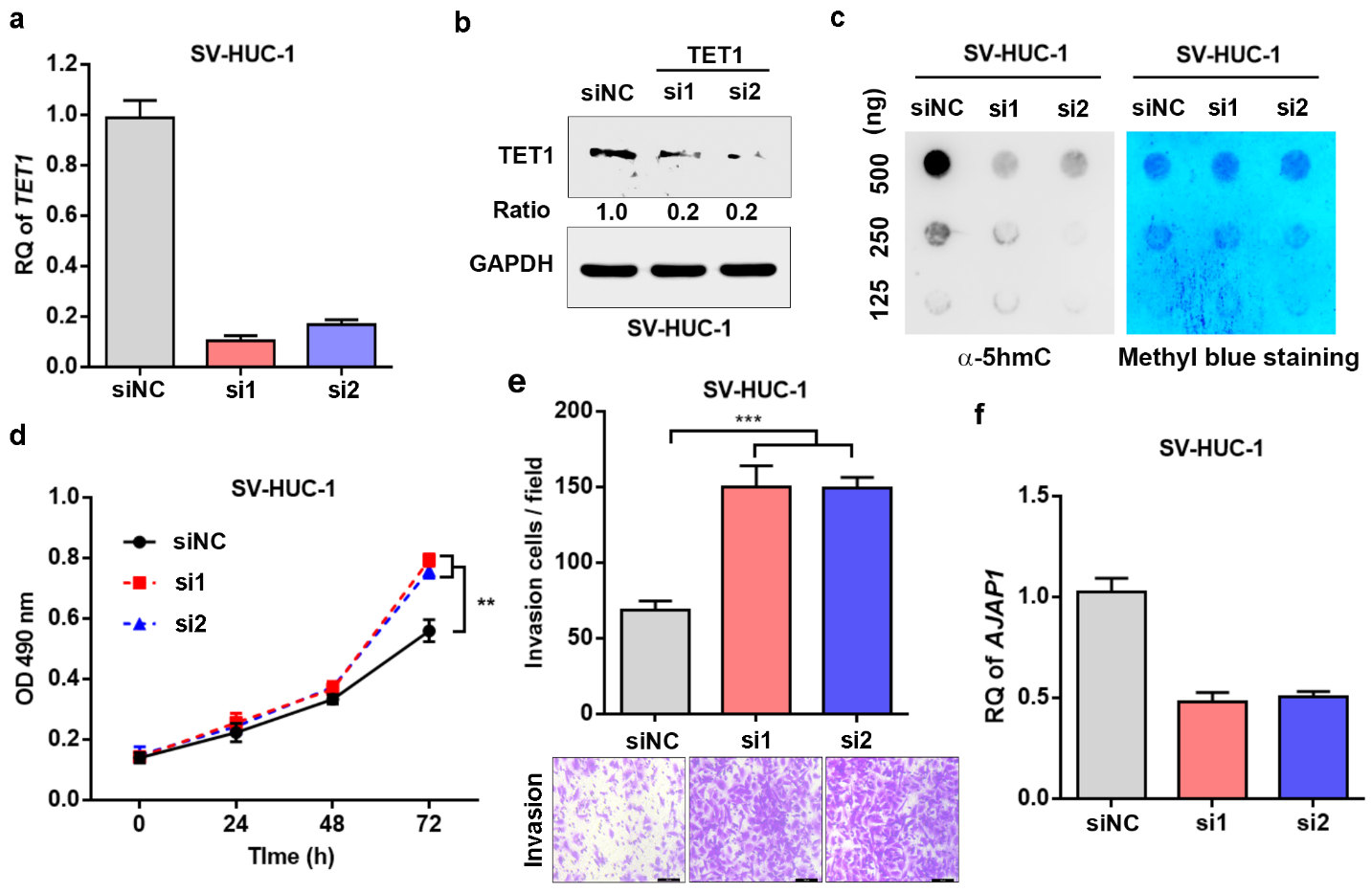


**Figure S2|** Knockdown of TET1 facilitates SV-HUC-1 cell proliferation and invasion. **(A, B)** The efficiency of knockdown of TET1 at mRNA **(A)** and protein (**B**) level, as well as 5hmC level **(C)** in SV-HUC-1 cells were confirmed by qRT-PCR, Western blotting and dot blot, respectively. **(D, E)** Effects of TET1 knockdown on cell proliferation for 72 h **(D)** and invasion for 20 h **(E)** of SV-HUC-1 cells by MTT and Transwell assays, respectively. Scale bar, 100 μm **(E)**. (**F**) The reduction of AJAP1 mRNA expression in TET1 knockdown SV-HUC-1 cells (si1 and si2) by qRT-PCR, compared with control cells (siNC). **, p < 0.01, ***, p < 0.001.

**
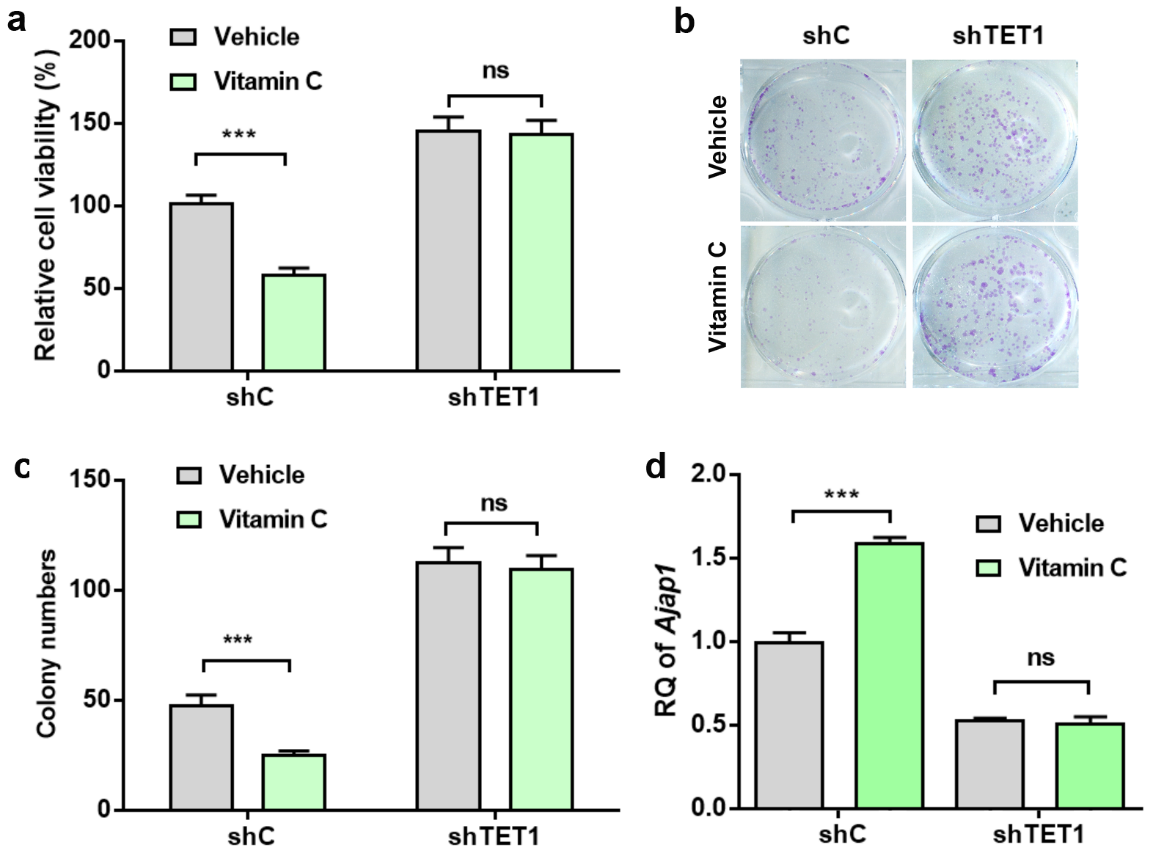
**

**Figure S3|** Inhibition of cell viability and induction of AJAP1 expression by vitamin C are dependent on TET1 expression. (**A**-**C**) Effects of 0.25 mM vitamin C on cell viability after 72 h (**A**) and colony formation for 10 d (**B**-**C**) among three different groups of 5637 cells. (**D**) The expression levels of AJAP1 in shC and shTET1 5637 cells with vehicle or 0.25 mM vitamin C treatment. shC, knockdown control cells; shTET1, TET1 knockdown 5637 cells. ***, p < 0.001; ns, not significant.

**
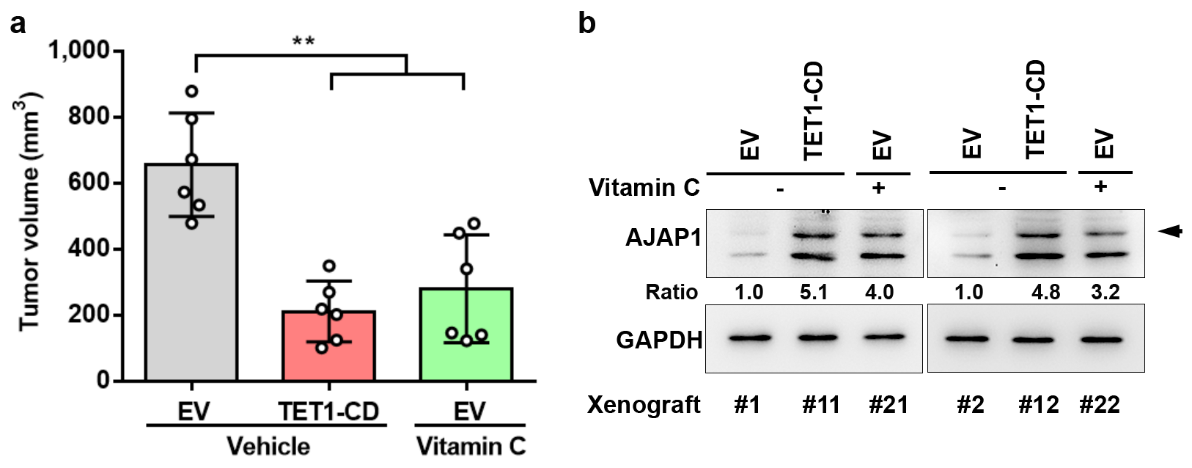
**

**Figure S4|** The ectopic expression of TET1-CD and vitamin C (4 g/kg/d) treatment suppressed tumor volume (**A**) and induced AJAP1 expression at protein level (**B**) in T24 xenograft tissues, respectively. EV, empty vector control transfectant. Arrow indicates the band of AJAP1. **, p < 0.01.

**
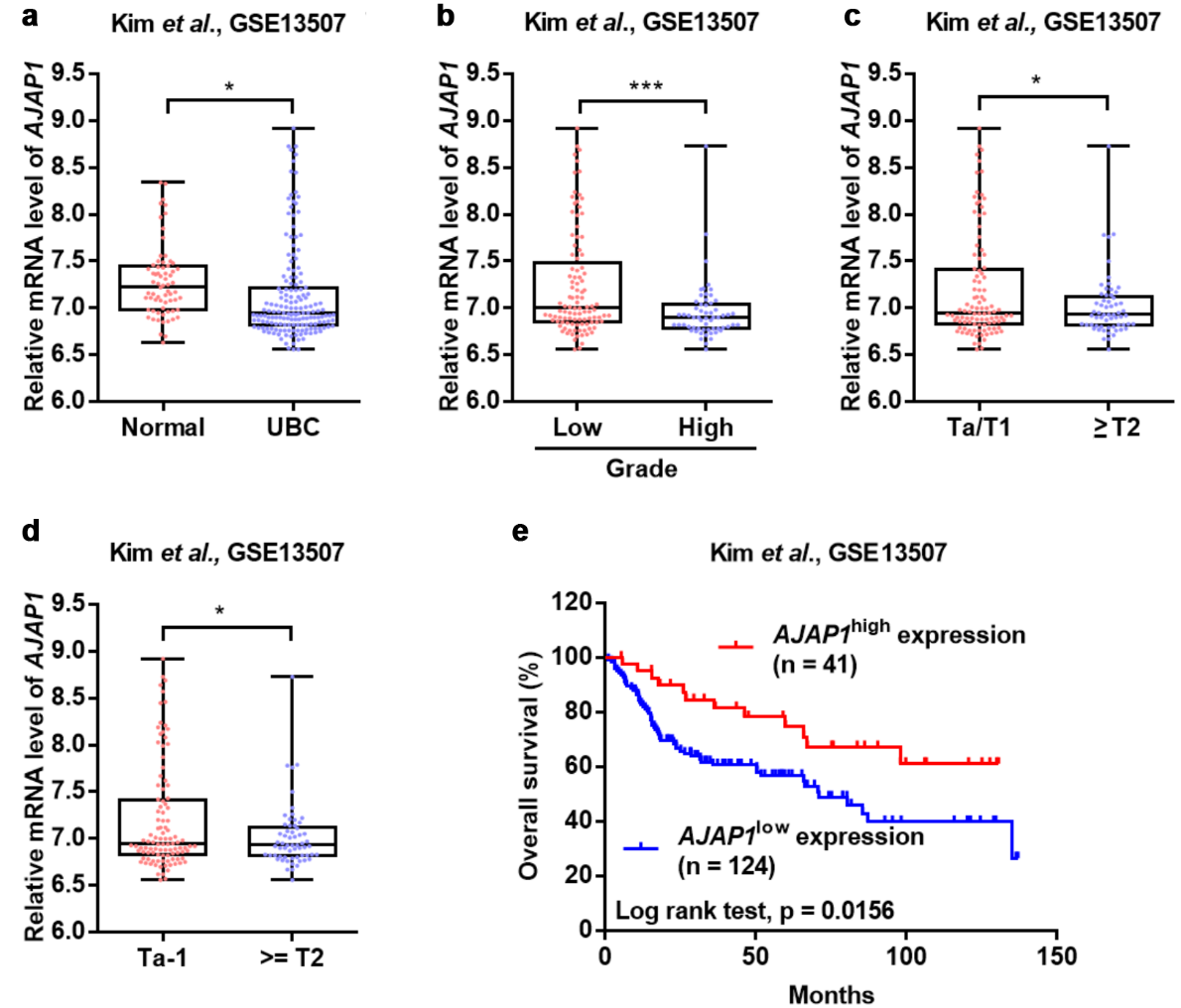
**

**Figure S5|** The mRNA expression levels of *AJAP1* in a database of human UBC specimens (GSE13507). (**A)** *AJAP1* is downregulated in human UBC samples (n = 165) compared with normal bladder mucosae surrounding cancer (n = 68). (**B-D)** The association of *AJAP1* expression with clinicopathological features, such as grade (**B**), stage (**C**) and superficial/invasive UBCs (**D**). (**E)** The overall survival rate of *AJAP1* expression in UBC patients (n = 165).

**
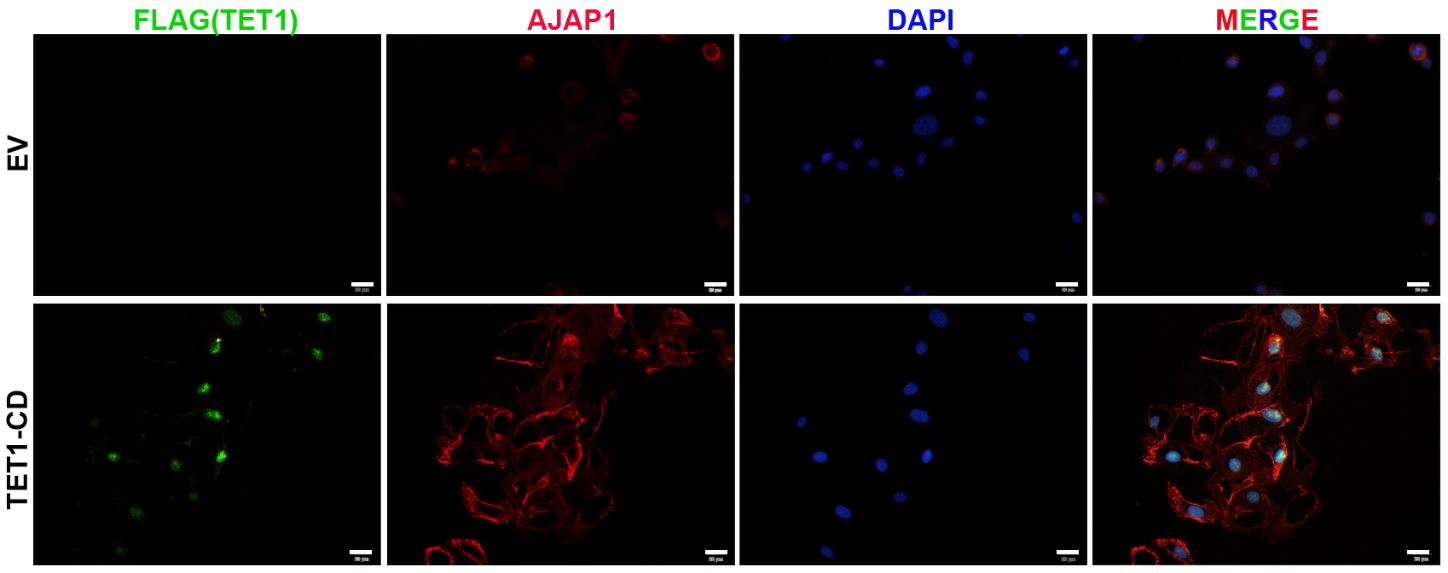
**

**Figure S6|** Overexpression of TET1-CD induces the expression level of AJAP1, which was detected on the cell membrane by immunofluorescence. The ectopic expression of TET1-CD was detected by the antibody to FLAG tag. DAPI was used to stain the nucleus. EV, empty vector control transfectants. Scale bar, 20 μm.

**Table S1**. List for qRT-PCR and hMeDIP primers, siRNAs and shRNAs.

|  | **Direction** | **Sequences (5’-3’)** |
| --- | --- | --- |
| **qRT-PCR Primers** |  |  |
| *β-actin* | Forward | CATGTACGTTGCTATCCAGGC |
|  | Reverse | CTCCTTAATGTCACGCACGAT |
| *AJAP1* | Forward | GAGACTGAGTTCATCGCCTGG |
|  | Reverse | CGTAAACGCCCGGAAATGTG |
| **hMeDIP** |  |  |
| hMeDIP-AJ-0-F | Forward | GCAAGCTGGCTGATGTGGTTT |
|  | Reverse | CAGGTAGAGCTGGAGGTCTGAC |
| hMeDIP-AJ-1-F | Forward | CTGGGCGATGAGGGACCGTT |
|  | Reverse | GCCTACGAGGGACTCCTCTGCT |
| hMeDIP-AJ-2-F | Forward | CTTCGCGTGCCATCCGGTTA |
|  | Reverse | CGGTCCCCACCTTGCGAA |
| hMeDIP-AJ-3-F | Forward | GGGGCATTCACCGAGCTCGT |
|  | Reverse | CGCAGTGCATCCCAGTGGA |
| **Subcloning for mammalian expression** | | |
| pTET1-CD-F | Forward | ACGATGACAAGCTTGCGGCCGCGGAACTGCCC  ACCTGCAGC |
| pTET1-CD-R | Reverse | TCTGCCCTCGATATCGAATTCGACCCAATGGTT  ATAGGGCCCC |
| pTET1CDmut-F | Forward | TCATCCC**T**ACAGGG**C**CATTCACAACATGAATA  ATGGAAGCAC |
| pTET1CDmut-R | Reverse | TGAATG**G**CCCTGT**A**GGGATGAGCACAGAAGTC  CAGGCAAGCA |
| **Subcloning for shRNA constructs** | | |
| shTET1-1F | Forward | CCGGGCAGCTAATGAAGGTCCAGAACTCGAG  TTCTGGACCTTCATTAGCTGCTTTTTG |
| shTET1-1R | Reverse | AATTCAAAAAGCAGCTAATGAAGGTCCAGAAC  TCGAGTTCTGGACCTTCATTAGCTGC |
| shTET1-2F | Forward | CCGGCCCAGAAGATTTAGAATTGATCTCGAG  ATCAATTCTAAATCTTCTGGGTTTTTG |
| shTET1-2R | Reverse | AATTCAAAAACCCAGAAGATTTAGAATTGATC  TCGAGATCAATTCTAAATCTTCTGGG |
| **siRNA sequences** | | |
| siNC |  | UUCUCCGAACGUGUCACGU |
| siAJAP1 |  | CCACAGAGACUGAGUUCAU |
| siTET1-1 |  | CCACAAAGUCACAUGAAUA |
| siTET1-2 |  | GAAAUAAUGAAGUGGAGUA |

**Table S2**. List of antibodies, chemicals and kits.

| **Antibody** | **Company** | **Cat. No.** | **Dilution (Application)** | **RRID** |
| --- | --- | --- | --- | --- |
| 5-hmC | Active Motif | 39769 | 1:10,000 (Dot Blot);  1:1,000 (IHC) | AB_10013602 |
| AJAP1 | Abcam | ab223117 | 1:1,000 (WB); 1:250 (IHC) | N.A. |
| Active β-catenin | Cell Signaling Technology | 8814s | 1:1,000 (WB) | AB_11127203 |
| β-catenin | Cell Signaling Technology | 9581s | 1:1,000 (WB) | AB_490891 |
| β-catenin | BD Biosciences | 610153 | 1:400 (IF); 1:500 (IHC) | AB_397554 |
| CD44 | Cell Signaling Technology | 3570s | 1:1,000 (WB) | AB_2076465 |
| Fibronectin | Santa Cruz | sc-8422 | 1:500 (WB) | AB_627598 |
| GAPDH | Santa Cruz | sc-32233 | 1:2,000 (WB) | AB_627679 |
| Lamin B1 | Cell Signaling Technology | 13435S | 1:1,000 (WB) | AB_2737428 |
| TET1 | GeneTex | GTX124207 | 1:1,000 (WB); 1:500 (IHC) | AB_11176491 |
| 2nd antibody mouse IgG | Cell Signaling Technology | 7076s | 1:5,000 (WB) | AB_330924 |
| 2nd antibody rabbit IgG | Cell Signaling Technology | 7074s | 1:5,000 (WB) | AB_2614860 |
| Alexa 555-conjugated 2nd antibody | Molecular  Probes | A-21428 | 1:1,000 (IF) | AB_141784 |
| **Chemicals** | | **Company** | **Cat. No.** |  |
| 5-aza-dC | | Sigma-Aldrich | A3656 |  |
| Crystal violet | | Sangon Biotech | A100528 |  |
| p-iodonitrotetrazolium violet | | Sigma-Aldrich | 18377 |  |
| Lipofectamine 3000 | | Thermo Fisher | L3000015 |  |
| Methylene blue Solution | | Sangon Biotech | A610622 |  |
| MTT | | Sigma-Aldrich | M2128 |  |
| Protein A/G plus-agarose | | Santa Cruz Biotechnology | sc-2003 |  |
| Puromycin | | Yeason | ISY1130 |  |
| TRIzol | | TaKaRa | 9109 |  |
| Vitamin C | | Sigma-Aldrich | A4034 |  |
| **Kits** | | **Company** | **Cat. No.** |  |
| Annexin V-FITC/PI double staining kit | | Yeasen | 40302 |  |
| Chromatin IP DNA Purification Kit | | Active Motif | 58002 |  |
| DAB kit | | Maixin Bio | DAB-0031 |  |
| ECL substrate kit | | Tanon | 180-501 |  |
| hMeDIP kit | | Active Motif | 55010 |  |
| Mut Express II Fast Mutagenesis Kit | | Vazyme | C214-01 |  |
| Nuclear and Cytoplasmic Protein Extraction Kit | | Yeasen | 20126ES50 |  |
| Prime-Script RT-PCR kit | | TaKaRa | RR047 |  |
| QIAamp DNA Mini Kit | | Qiagen | 51304 |  |
